# Osteoma in a Domestic Goose: Radiological and Histopathological Evaluation

**DOI:** 10.3390/ani15070942

**Published:** 2025-03-25

**Authors:** Michał Gesek, Adrianna Michniewicz, Ewa Łukaszuk

**Affiliations:** 1 Department of Pathological Anatomy, Faculty of Veterinary Medicine, University of Warmia and Mazury in Olsztyn, 10-719 Olsztyn, Poland; adrianna.michniewicz@student.uwm.edu.pl; 2 Department of Poultry Diseases, Faculty of Veterinary Medicine, University of Warmia and Mazury in Olsztyn, 10-719 Olsztyn, Poland; ewa.lukaszuk@uwm.edu.pl

**Keywords:** osteoma, goose, tumour

## Abstract

The aim of this work was a detailed radiological and histopathological evaluation of a solid tumour that was diagnosed in a 2-year-old goose (*Anser domesticus*). The radiograph examination showed an osseous change involving the cervical vertebrae. The tumour measuring 15 cm × 10 cm × 9 cm was dense and had well-defined borders, suggesting the presence of calcified bone tissue. Histopathology revealed a well-defined benign neoplasm derived from bone that consisted largely of irregular, disorganized bone trabeculae surrounded by a layer of osteoblasts. The tumour has been classified as an osteoma, which originates from the body of the vertebrae. Osteoma is a benign, well-differentiated tumour with a structure that resembles bone tissue. It presents as a well-demarcated, hard, single tumour that can grow to a considerable size. The aetiology of osteomas in birds remains unclear because of the small number of cases described. Therefore, the influence of factors such as age, breed or sex, trauma, embryonic malformation, infection, developmental disorders, and genetic factors on the development of this type of tumour has not been established. Trauma seems to be the most obvious cause of growth in this case. This work provides valuable information about osteomas in birds, which is important for understanding such neoplasms.

## Simple Summary

A solid tumour was detected in a 2-year-old goose (*Anser domesticus*). The radiographic examination showed an osseous change involving the cervical vertebrae. The tumour measuring 15 cm × 10 cm × 9 cm was hard, suggesting the presence of calcified bone tissue. Histopathology revealed a well-defined benign neoplasm derived from bone that consisted largely of irregular, disorganized bone trabeculae. The tumour has been classified as an osteoma, originating from the body of the vertebrae. Osteoma is a benign, well-differentiated tumour with a structure that resembles bone tissue. Trauma seems to be the most obvious cause of growth in this case.

## 1. Introduction

Neoplasms in birds are mainly found in wild birds, pet birds, exotic birds and, to a lesser extent, in farming poultry [1]. Tumours described in birds include descriptions of neoplasms of the skin, internal organs (liver, kidney), reproductive system, brain, vascular system, connective tissue, bones, and eyes. Neoplastic diseases of the skeletal system are rare (6.66%), and the available literature is limited [1]. Bone tumours include osteomas and osteosarcomas; in the available literature, there have been descriptions of osteomas in peach-faced lovebirds [2,3], blue-fronted amazon parrots [4], owls [5,6], canaries [1,7], hens [8], broiler chickens [9], domestic ducks, budgerigars [1,10], and eclectus parrots [11].

Osteosarcomas have been described in brown leghorn hens [12], a 30-week-old hen [8], free-range chickens [13], broiler chickens [9], Japanese quail, cockatoos, doves [10], budgerigars, mallards [1], and the foot of a goose [14].

Benign neoplasia may originate from the tarsometatarsus, plantar footpad, cranium [10], scapula, wing [2,5,6,7], nasal cavity [4], cutis [3], long bone [9], and sternum [11].

Bone tumours are very rarely described in breeding birds due to the short fattening period or the large number of birds in breeding flocks where proliferative changes may be overlooked. Neoplasia in geese is still a poorly understood and described area of avipathology and therefore an area for further analysis. The case of a tumour in a two-year-old gander presents interesting research material that could contribute to the knowledge and development of methods for diagnosing tumours in birds. The main suspicion is that trauma was the cause of the growth of the tumour.

The aim of this work is the radiological and histopathological evaluation of a solid lesion diagnosed in a goose, which is suspected to be neoplastic in nature.

## 2. Materials and Methods

During a clinical examination of a goose farm, the veterinarians noticed the presence of a 2-year-old male showing symptoms of a disorder. The clinical examination revealed the presence of a large mass in the neck and spine, which probably contributed to a neurological disorder (twisting of the neck from side to side—torticollis). The clinicians, together with the breeder who had given his consent, decided to euthanise the bird on humanitarian grounds—prior sedation with 2 mL of intramuscular ketamine (Bioketan Vetoquinol, Gorzow Wielkopolski, Poland) and then an intracardiac overdose of anaesthesia, with pentobarbital sodium and phenytoin sodium (EUTHASOL^®^ Virbac, Carros, France). All euthanasia procedures complied with Directive 2010/63/EU of the European Parliament and of the Council of 22 September 2010 on the protection of animals used for scientific purposes, as well as the Polish Animal Welfare Act of 21 August 1997. Then, the clinicians secured the body and transferred it for further research. The carcass of the male goose (*Anser domesticus*) was delivered to the Department of Pathological Anatomy at the University of Warmia and Mazury in Olsztyn. A radiological examination and a detailed necropsy were carried out. The internal organs were fixed in 10 % formalin and the tumour was removed, fixed in 10% formalin, and decalcified. The collected and fixed specimens were then subjected to routine histopathological examination (haematoxylin and eosin staining was performed—HE). In addition, tumour samples were stained with Mallory Trichrome for connective tissue (Mallory Trichrome, Bio-Optica, Milano, Italy).

## 3. Results

### 3.1. Results of Radiological Examination

**Figure 1:**
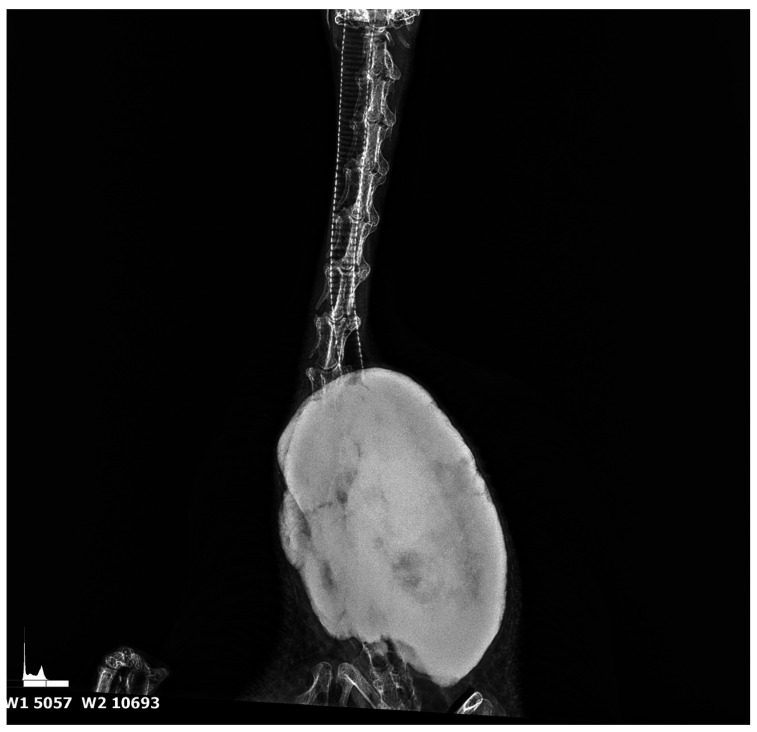
Ventral–dorsal radiograph of the cervical region in goose. Large hyperechogenic mass tightly fused to the spine—derived from the vertebrae.

**Figure 2:**
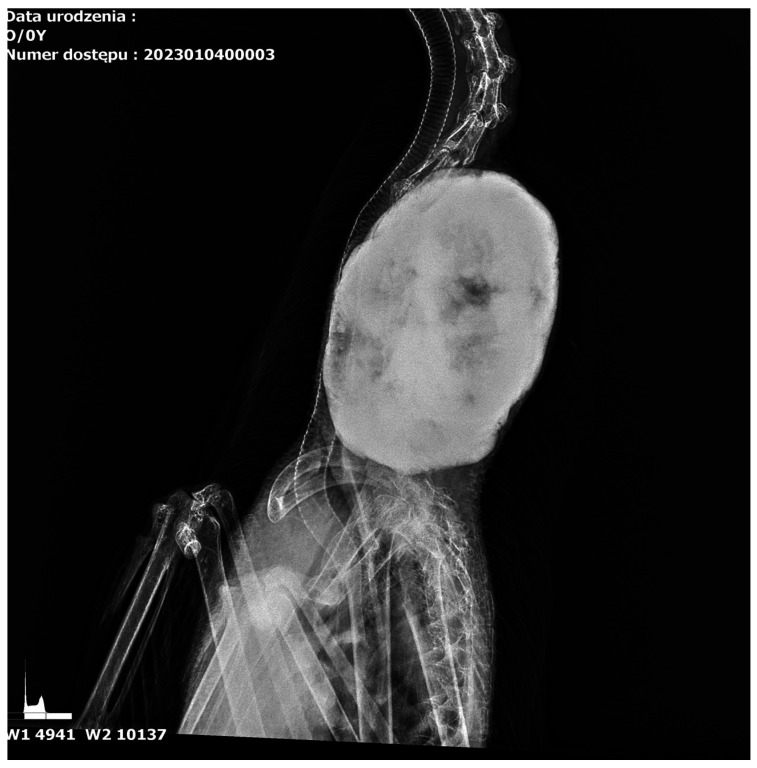
Lateral radiograph of the cervical region in goose. The tumour pushed the trachea to the sides of the neck without altering its patency.

The radiograph examination was taken in two projections—dorsal and lateral (Figure 1 and Figure 2)—and in the area of the 8–13 cervical vertebrae; an osseous change involving other cervical vertebrae was noted. The change was a well-circumscribed hyperechogenic mass extending into the thoracic cavity. The tumour measuring 15 cm in length and 10 cm in width was dense and had well-defined borders, suggesting the presence of calcified bone tissue. The tumour was spherical with a homogeneous surface, indicating slow and mild growth. It showed signs of calcification without causing visible destruction of the surrounding soft tissues and adjacent structures. The tumour slightly pushed the trachea and oesophagus at the sides of the neck but did not affect their patency.

### 3.2. Results of Macroscopic Examination

**Figure 3:**
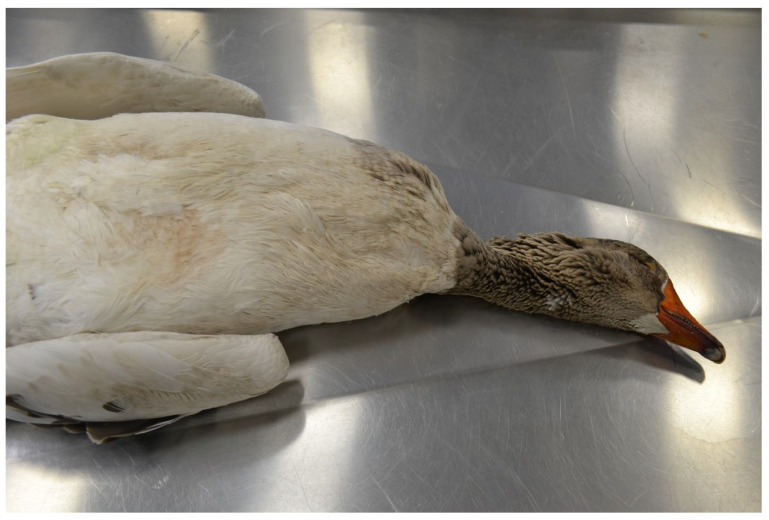
Domestic goose. Large tumour in the distal part of the cervical region without skin lesions and ulcerations.

**Figure 4:**
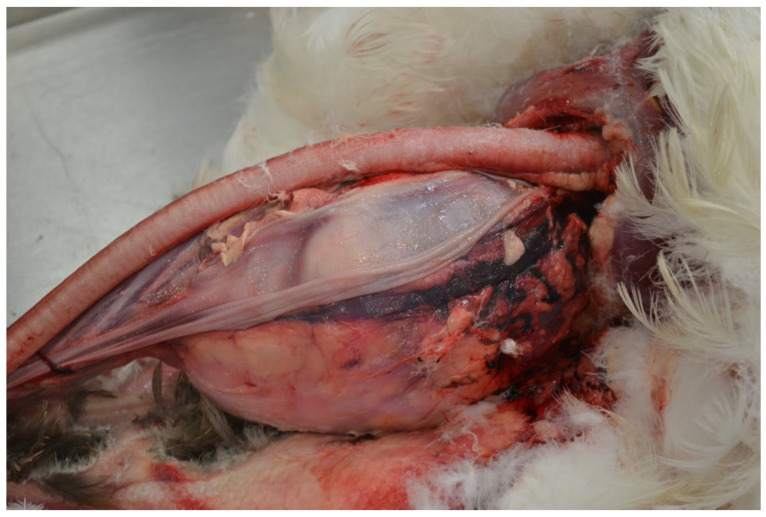
Domestic goose. The well-demarcated tumour pushed the trachea and oesophagus to the sides of the neck without altering its patency.

**Figure 5:**
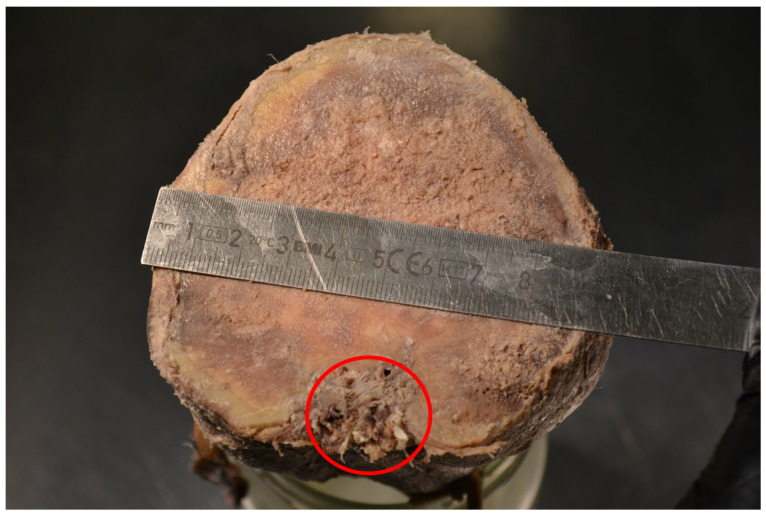
Domestic goose. The cross-section of the tumour with visible presence of cervical vertebrae (circle).

An autopsy revealed a solid tumour measuring 15 cm × 10 cm × 9 cm, located in the cervical vertebrae (Figure 3). The tumour extended into the thoracic cavity (Figure 4), weighed 2 kg, and consisted of hard, compact bone tissue that was tightly fused to the spine, originating from the 8–13 cervical vertebrae (Figure 5). There was no evidence of metastasis of the bone tumour to other organs and tissues. Despite its size, the tumour did not infiltrate the trachea or oesophagus but moved them to the side of the neck. The surface of the tumour was slightly rough, but the surrounding soft tissue and skin showed no signs of infiltration. The macroscopic evaluation of the organs and tissues did not reveal any pathological changes.

### 3.3. Results of Microscopic Examination

**Figure 6:**
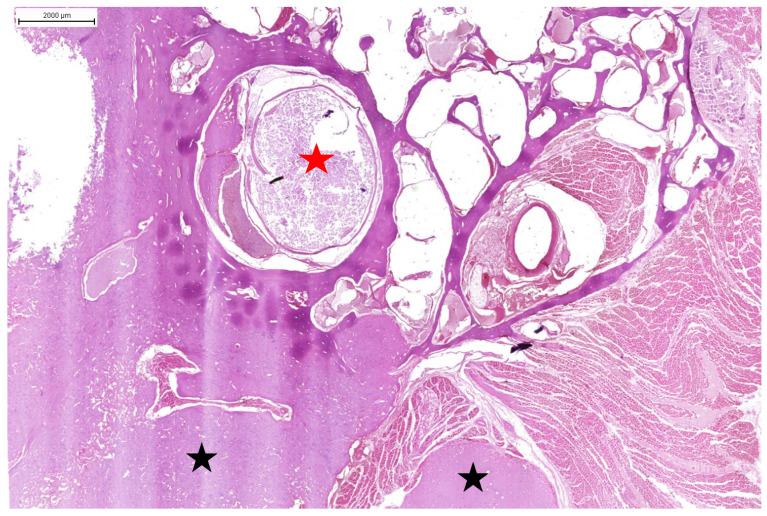
Domestic goose. HE staining of cervical vertebrae with tumour. Visible central canal with spinal cord (red asterisk). Below that is a visible tumour mass originating from the body of the cervical vertebrae (black asterisks).

**Figure 7:**
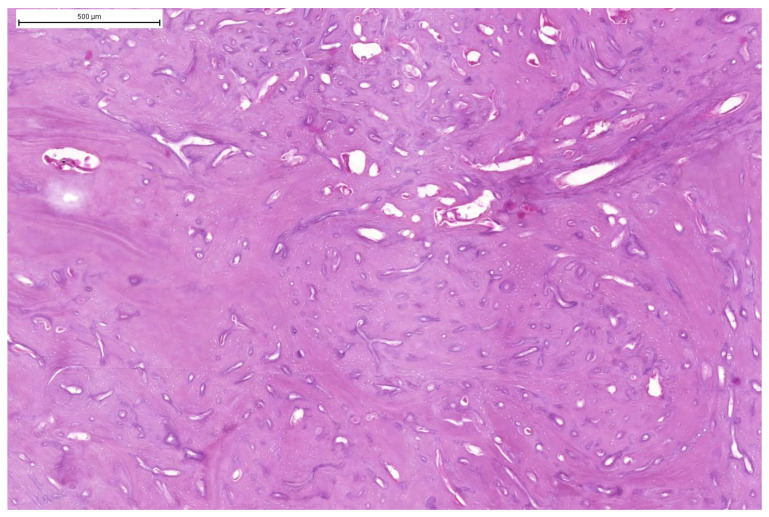
Domestic goose. HE staining of tumour mass. Large dense area of trabecular bone with visible irregular holes containing blood vessels and neoplastic osteoblasts.

**Figure 8:**
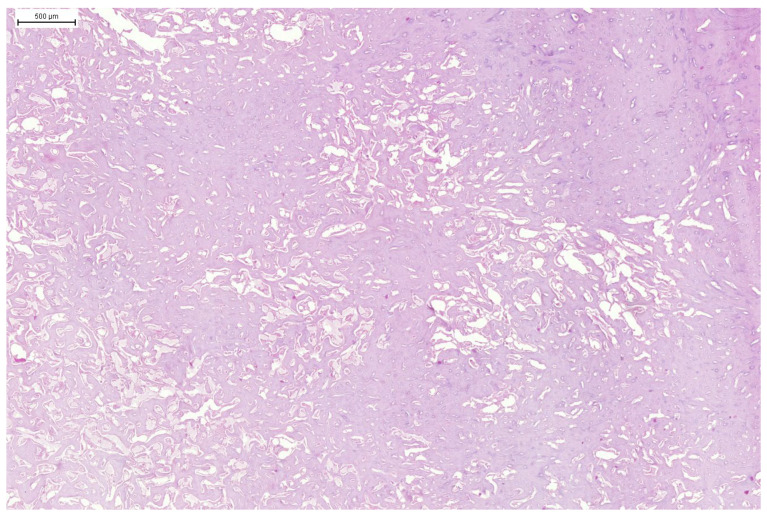
Domestic goose. HE staining of tumour mass. Large area of trabecular bone with visible irregular holes containing blood vessels and osteoblasts.

**Figure 9:**
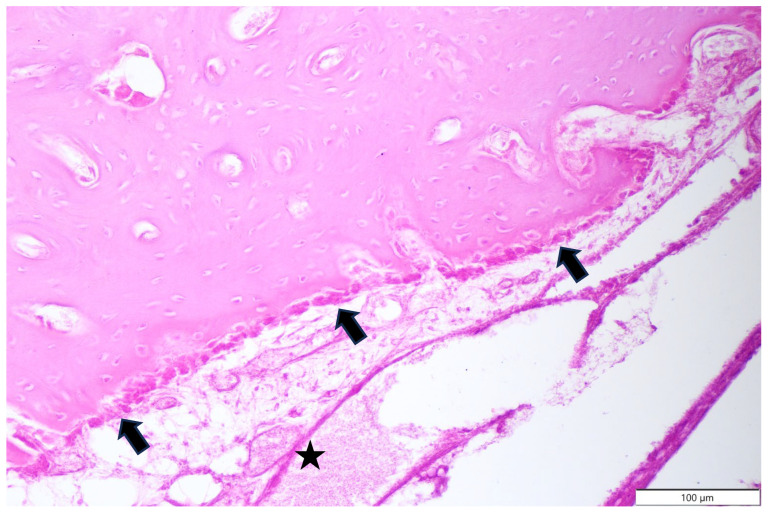
Domestic goose. HE staining of tumour mass. Single layer of osteoblasts (arrows) forming trabecular bone. Connective tissue (asterisk).

**Figure 10:**
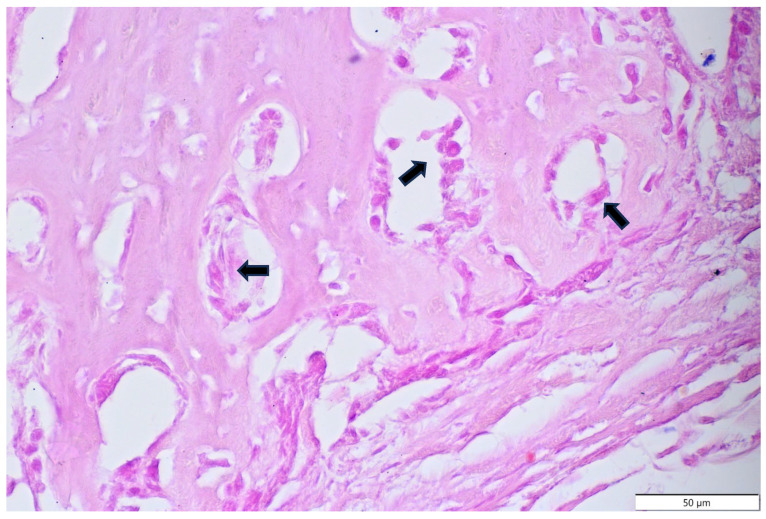
Domestic goose. HE staining of tumour mass. Single layer of osteoblasts (arrows) forming trabecular bone.

**Figure 11:**
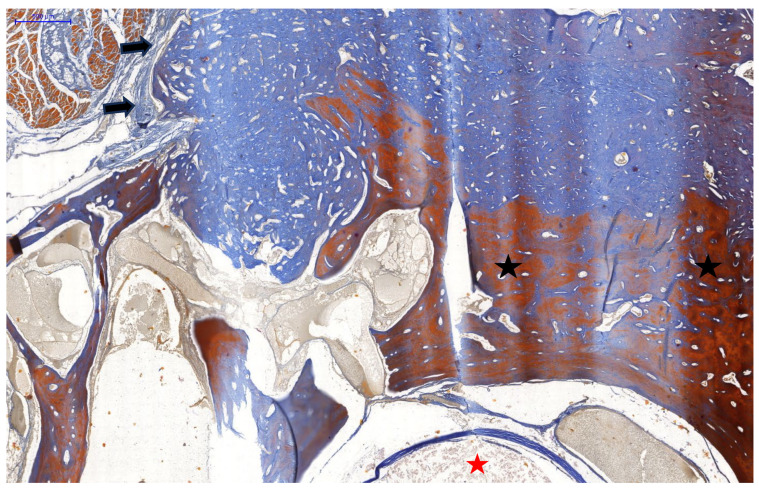
Domestic goose. Mallory trichrome staining of tumour mass and cervical vertebrae with tumour. Visible central canal with spinal cord (red asterisk). Collagen-rich (blue colour) tumour mass arising from the body of the cervical vertebrae (black asterisks). Connective tissue (arrows) separating tumour from cervical muscles.

Histopathological examination confirmed the presence of a well-defined benign neoplasm derived from bone tissue, which arose from the body of the cervical vertebrae (Figure 6). The tumour was well circumscribed and consisted largely of irregular, disorganized, immature lamellar bone, surrounded by a single layer of non-reactive well-differentiated osteoblasts (Figure 7, Figure 8, Figure 9 and Figure 10). Small intertrabecular spaces contained small amounts of haematopoietic tissue, osteoblasts, and blood vessels (Figure 9). The tumour was demarcated from the cervical muscles and lined with connective tissue (Figure 11). A histopathological examination of the tumour showed that the change was mild and originated from bone tissue. The tumour was classified as a central osteoma arising from the endosteum, due to the tumour’s growth from the vertebral body and the lack of an obvious inciting cause. Mallory Trichrome staining revealed an immature, collagen-rich tumour mass arising from the ossified endosteum of the cervical vertebrae (Figure 11). The microscopic evaluation of the organs (lungs, liver) did not reveal any pathological changes or metastasis.

## 4. Discussion

Radiological and histopathological examination revealed the presence of an osteoma. To the best of our knowledge, this is the first case of a benign bone tumour originating from the cervical vertebrae. Benign bone cancers are rare in birds, and the aetiology is poorly understood [1].

An osteoma is a benign, well-differentiated tumour with a structure that resembles bone tissue [15,16]. It usually presents as a well-demarcated, hard, single tumour that can grow to a considerable size [15,16]. Osteomas are characterised by slow growth that can last for many months, after which the tumour reaches a large size and often stops growing [15]. These tumours can remain dormant for a long time, often for years, without causing any clinical symptoms, until the tumour grows again [15].

The present case describes a large tumour; notably, the previous cases of osteomas in birds have not reported growth to this size. The osteoma described in the left nostril of a 24-year-old amazon parrot was 5 mm in diameter, and a total excision of the lesion resulted in complete recovery [4]. Cowan et al. [11] described interesting findings in an eclectus parrot, where an osteoma that was 1 cm in diameter was diagnosed in the sternum. The tumour exhibited well-differentiated trabecular bone containing osteocytes, osteoblasts, and osteoclasts, with spaces of bone marrow. The case of an osteoma in the left wing of a canary reached a size of 12 × 8 × 6 mm, and the histopathological changes did not demonstrate such wide areas of osseous trabeculae as in our case [7]. Cagnini et al. [2] showed an osteoma in the same location but with larger dimensions (22 × 20 × 22 mm), and the histological image revealed a wider area of mature trabeculated bone formation. In a lovebird, bilateral cutis osteomas at the junction of the ulnar radius and humerus, measuring 3.4 × 2.2 cm and 2.4 × 2.7 cm, showed bone spicules with osteoblastic/osteocytic proliferation, along with the presence of mineralised woven bone matrix. Together with the tumour content, active inflammatory changes with cellular infiltrations were noted [3]. No inflammatory changes were diagnosed in our study. Hahn et al. [6] described a case of an osteoma in a barred owl, with a larger size on the left radius (7 × 5 × 4.5 cm). The body of the tumour consisted of multiple large, coalescing islands of well-differentiated cartilage separated by small spicules of mature bone or thin connective tissue. The presence of the tumour caused muscle atrophy and skin ulceration. In our case, such changes were not seen. A tumour of similar size was reported in a great horned owl (5 × 4.5 × 3.5 cm) and was attached to the left ulna [5]. Histopathological examination revealed thick trabeculae of lamellar and woven bone from the periosteum, supported by abundant loose fibrovascular stroma. After the tumour was completely resected, there was no evidence of regrowth. Although our described tumour was large, it did not exert pressure on the spinal cord; instead, it grew, compressed the surrounding tissues, and displaced other organs (trachea, oesophagus), but did not cause a direct threat to life. In addition, no atrophic changes and no inflammatory response were observed in the tissues that were examined.

The aetiology of osteomas in birds remains unclear, mainly because of the small number of cases described. Various authors have pointed out that the cause is still unclear, and therefore the influence of factors such as age, breed or sex, trauma, embryonic malformation, infection, developmental disorders, and genetic factors on the development of this type of tumour has been suggested [1,4,6,7]. More reliable information has been provided in the work of Cowan et al. [11], in which trauma was the main cause of an osteoma in the eclectus parrot. Three months after the injury, a tumour was found in the sternum. Similar observations have been confirmed by Pinzon-Osorio et al. [3], where in the lovebird studied, owner-reported traumatic wing injuries (1 month prior) were the most likely cause of tumour growth. Considering the case described, the necks of birds—especially geese—can be long and uncovered, and it is very likely that an injury was the cause of the growth of the current tumour. We do not know if other birds were involved or if there was a human factor, e.g., mistreatment of birds on the farm or a deliberate attack. In the early stages, a tumour grows slowly and can only be diagnosed by radiographic examination in a small number of cases when it causes visible clinical symptoms or when it is visible. In the case of pet birds that are in direct contact with their owners, even minor changes and clinical signs may be observed at an earlier stage. In farm animals, such changes are rarely seen, and therefore tumours can grow rapidly at a later stage, which has been confirmed in other studies [6]. This confirms our suspicion that, in the 2-year-old gander examined, the trauma may have occurred much earlier, perhaps even at a young age, and the tumour may have started to grow over time.

## 5. Conclusions

Bone-derived neoplasms not associated with infectious factors are rare in aviopathology. Confirming the clinical occurrence of an osteoma in a domestic goose can be valuable for further studies in this field.

## Data Availability

Data will be provided on request.
